# HLA-B*13, B*35 and B*39 Alleles Are Closely Associated With the Lack of Response to ART in HIV Infection: A Cohort Study in a Population of Northern Brazil

**DOI:** 10.3389/fimmu.2022.829126

**Published:** 2022-03-16

**Authors:** Leonn Mendes Soares Pereira, Eliane dos Santos França, Iran Barros Costa, Erika Vanessa Oliveira Jorge, Patrícia Jeanne de Souza Mendonça Mattos, Amaury Bentes Cunha Freire, Francisco Lúzio de Paula Ramos, Talita Antonia Furtado Monteiro, Olinda Macedo, Rita Catarina Medeiros Sousa, Eduardo José Melo dos Santos, Felipe Bonfim Freitas, Igor Brasil Costa, Antonio Carlos Rosário Vallinoto

**Affiliations:** ^1^ Virology Laboratory, Institute of Biological Sciences, Federal University of Pará, Belém, Brazil; ^2^ Epstein-Barr Virus Laboratory, Virology Unit, Evandro Chagas Institute, Ananindeua, Brazil; ^3^ Department of Immunogenetics, Hemotherapy and Hematology Foundation of the State of Pará, Belém, Brazil; ^4^ Epidemiology and Surveillance Service, Evandro Chagas Institute, Ananindeua, Brazil; ^5^ Retrovirus Laboratory, Virology Unit, Evandro Chagas Institute, Ananindeua, Brazil; ^6^ School of Medicine, Federal University of Pará, Belém, Brazil; ^7^ Laboratory of Human and Medical Genetics, Institute of Biological Sciences, Federal University of Pará, Belém, Brazil; ^8^ Graduate Program in Biology of Infectious and Parasitic Agents, Institute of Biological Sciences, Federal University of Pará, Belém, Brazil

**Keywords:** HIV, therapeutic response, immunogenetics, HLA, B*13

## Abstract

**Introduction:**

Immune reconstitution failure after HIV treatment is a multifactorial phenomenon that may also be associated with a single polymorphism of human leukocyte antigen (HLA); however, few reports include patients from the Brazilian Amazon. Our objective was to evaluate the association of the immunogenic profile of the “classical” HLA-I and HLA-II loci with treatment nonresponse in a regional cohort monitored over 24 months since HIV diagnosis.

**Materials and Methods:**

Treatment-free participants from reference centers in the state of Pará, Brazil, were enrolled. Infection screening was performed using enzyme immunoassays (Murex AG/AB Combination DiaSorin, UK) and confirmed by immunoblots (Bio-Manguinhos, FIOCRUZ). Plasma viral load was quantified by real-time PCR (ABBOTT, Chicago, Illinois, USA). CD4^+^/CD8^+^ T lymphocyte quantification was performed by immunophenotyping and flow cytometry (BD Biosciences, San Jose, CA, USA). Infection was monitored *via* test and logistics platforms (SISCEL and SICLOM). Therapeutic response failure was inferred based on CD4^+^ T lymphocyte quantification after 1 year of therapy. Loci A, B and DRB1 were genotyped using PCR-SSO (One Lambda Inc., Canoga Park, CA, USA). Statistical tests were applied using GENEPOP, GraphPad Prism 8.4.3 and BioEstat 5.3.

**Results:**

Of the 270 patients monitored, 134 responded to treatment (CD4^+^ ≥ 500 cells/µL), and 136 did not respond to treatment (CD4^+^ < 500 cells/µL). The allele frequencies of the loci were similar to heterogeneous populations. The allelic profile of locus B was statistically associated with treatment nonresponse, and the B*13, B*35 and B*39 alleles had the greatest probabilistic influence. The B*13 allele had the highest risk of treatment nonresponse, and carriers of the allele had a detectable viral load and a CD4+ T lymphocyte count less than 400 cells/µL with up to 2 years of therapy. The B*13 allele was associated with a switch in treatment regimens, preferably to efavirenz (EFZ)-based regimens, and among those who switched regimens, half had a history of coinfection with tuberculosis.

**Conclusions:**

The allelic variants of the B locus are more associated with non-response to therapy in people living with HIV (PLHIV) from a heterogeneous population in the Brazilian Amazon.

## Introduction

In contrast to what was expected with the introduction of antiretroviral therapy (ART), treatment maintenance fails to restore the CD4^+^ T lymphocyte count in a portion of people living with HIV (PLHIV) and consequently enables immune dysfunction and observed morbidities when compared to complete immune reconstitution (1).

There is no consensus regarding the definition of “treatment nonresponders”. However, the prevailing understanding is that an adequate immune response should include a CD4^+^ T lymphocyte count greater than 500 cells/μL because the morbidity rate for individuals with this immune profile is similar to that for seronegative individuals (2).

The mechanisms associated with immune reconstitution failure are varied and, in most cases, dependent; these mechanisms manifest as dysfunctions in the balance of lymphocyte maintenance (3, 4). Among the set of associated factors, it is suggested that the genetic profile of key immune response elements may impact recovery failure (5).

The HLA system plays roles in various stages of HIV infection, i.e., the selection of the viral epitope repertoire presented to CD4^+^ and CD8^+^ T lymphocytes (6), the induction of antiviral cytotoxicity (7) and the selection of mutations in the virus, thus modulating viral escape kinetics (8).

In an overview, the alleles HLA-A*24 (9), A*36 (10), B*35 (11), B*53 (12), and DRB1*13 (13) are some associated with the progression of HIV infection. In contrast, the HLA-A*02 (14), B*27 (15), B*57 (16) and DRB1*15 (17) are examples associated with infection control. Rare alleles can lead to additional viral evolution compared to the more common alleles, particularly with respect to the HIV-1 *env, nef* and *pol* genes (18). On the other hand, heterozygosity is considered a selective advantage against the progression of infection due to the ability of the host to present a larger repertoire of viral antigens, thus developing a broader response (19).

In the maintenance of the therapeutic response, studies suggest that allelic variants of HLA may be associated with post-ART immune failure; for example, having the HLA-B*57 allele impairs the recovery of CD4^+^ T cells during the first three years after the onset of ART, even though the allele provides a potential mechanism for epidemiological protection against infection (20). However, discussions already point to the inability of ART to reverse the immune escape process of HIV associated with the HLA profile (21); for patients with the B*57 allele, immune escape may be aggravated by a hypersensitivity reaction to some antiretroviral drugs (22).

The allele clusters Bw4 and Bw6, which interact with killer cell immunoglobulin-like receptors (KIR), have been associated with HIV disease progression control as well as with resistance to infection in different populations (23, 24). In an Australian cohort, Bw4 homozygosity seems to predict a compromised recovery of CD4^+^ T lymphocytes after the start of combination ART (25). In a similar ethnic population, post-ART counts of CD4^+^ Bw4^+^ T lymphocytes were associated with the partial normalization of NK-KIR3DL1 cell functionality. However, the reconstitution of immune competence was not associated with the complete restoration of antibody-dependent cellular cytotoxicity (26).

Other class 1 and class 2 molecules are abnormally expressed in patients receiving ART, each related to an aspect of the immune response (27, 28). It is likely that in this context, some alleles may mediate post-ART immune failure (29); however, others are camouflaged by the effects of therapy (30).

In this context, we observed a lack of studies on the relationship between HLA and treatment nonresponse in patients from the Brazilian Amazon and the northern region of Brazil. In Brazil, the frequency of nonresponders tends to vary from 38 to 50% depending on the region and on the methodology used in studies, in which biomarkers of varying etiology have been associated with the observed immunological condition (31–33).

Thus, herein, we evaluate the frequency and possible associations of HLA class 1 (HLA-A and HLA-B) and class 2 (HLA-DRB1) alleles in nonresponder PLHIVs from a northern Brazilian cohort.

## Materials and Methods

### Sampling and Ethical Aspects

This cohort study was created by recruiting patients from *Setor de Atendimento Médico Unificado do Instituto Evandro Chagas* (Unified Medical Service of the Evandro Chagas Institute; SOAMU-IEC) and *Centro de Atenção à Saúde nas Doenças Infecciosas Adquiridas* (Health Care Center for Acquired Infectious Diseases; CASA DIA) through the weekly selection of peripheral blood samples from prescreened PLHIV or suspected HIV-infected people living in the state of Pará, Brazil, from January 2018 to January 2019.

In compliance with resolutions 466/2012 and 347/05 of the National Health Council, which addresses the guidelines and regulatory standards of research involving human beings, the project was submitted for ethical review and approved by the Human Research Ethics Committee of *Instituto Evandro Chagas* (Protocol: 3.121.265; CAAE: 73927717.3.0000.0019). All participants were informed about the study objectives, and those who agreed to participate signed an informed consent form and answered a sociodemographic questionnaire. Individuals under 18 years of age or who were already receiving ART when first approached were not considered for the study. Participants who died, those who did not have continuous data on monitoring platforms, or those who abandoned the governmental assistance program for PLHIV during the study period were excluded.

### Confirmatory Methods, Quantification of Viral Load and CD4^+^ and CD8^+^ T Lymphocytes

The suspicion of HIV infection was confirmed by the qualitative detection of p24 antigen and anti-HIV-1 and anti-HIV-2 IgG antibodies by enzyme immunoassays (Murex AG/AB Combination DiaSorin, UK). Serology was confirmed using a rapid Immunoblot DPP HIV-1/2 kit (Bio-Manguinhos, FIOCRUZ) following the manufacturer’s protocol.

Plasma HIV viral load was quantified by real-time PCR using an Abbott mSample Preparation System RNA extraction kit and Abbott Real-Time HIV-1 amplification matrix (ABBOTT, Chicago, Illinois, USA).

The quantification of CD4 T (CD45^high^ CD3^+^ CD4^+^ CD8^-^) and CD8 T (CD45^high^ CD3^+^ CD4^-^ CD8^+^) lymphocytes was performed by immunophenotyping and flow cytometry using BD FACSCalibur-4 core equipment, FACSCount™ Reagents and Tritest™/TruCount kits (BD Biosciences, San Jose, CA, USA).

### Monitoring of Infection and Grouping Into Therapeutic Response Profiles

Access to the monitoring data platforms was obtained through pre-established authorizations declared for ethical review and in accordance with the consent of the participants.

Lymphocyte quantification and viral load data, after the first collection, were obtained from *Sistema de Controle de Exames Laboratoriais da Rede Nacional de Contagem de Linfócitos CD4^+^/CD8^+^ e Carga Viral do HIV* (Laboratory Test Control System of the National Network of CD4^+^/CD8^+^ Lymphocyte Count and HIV Viral Load; SISCEL) every six months. The quantification methods in each period were the same as previously described. The data regarding the management and distribution of antiretrovirals were obtained from *Sistema de Controle Logístico de Medicamentos* (Medication Logistics Control System; SICLOM) every six months; the initial therapeutic regimens, changes in approach and reasons for changes were determined. The data collection occurred for twenty-four months from the date of inclusion in the study.

In total, 270 PLHIVs participated in the study; after one year of ART, these PLHIVs were stratified based on their immune response to treatment into treatment nonresponders (CD4^+^ < 500 cells/µL; varied viral load) and treatment responders (CD4^+^ ≥ 500 cells/µL; varied viral load). We chose this cutoff because a previous analysis showed that for the same period, the viral load itself was not a determining factor in the differentiation of different progression groups. These established groups were evaluated for another year of therapy, totaling 24 months of analysis and data collection.

### DNA Extraction and Genotyping of the HLA Locus

Peripheral blood samples were collected from the participants, from which DNA was extracted using a QIAamp DNA Mini Kit (Qiagen, Düsseldorf, Nordrhein-Westfalen, Germany) following the manufacturer’s recommendations. The newly extracted DNA samples were quantified using a Qubit spectrofluorometer (Invitrogen, USA), following the manufacturer’s recommendations; and the degree of purity was evaluated using a NanoDrop™ 2000/2000c spectrophotometer (Waltham, Massachusetts, USA), in which the elution solution used in the extraction of genetic material was used as a reference standard.

We standardized the following profiles as the ideal range for successful amplification: for loci A and DRB1, a concentration between 10 and 15 µg/ml; for the B locus, a concentration between 10 and 12 µg/mL. The degree of purity for all loci was represented by the ratios 260/280: 1.8-2.0 and 260/230: 1.8-2.2. Samples having concentrations above the established standard were diluted in DNase/RNase-free ultrapure distilled water (Invitrogen, USA). Samples with concentrations below the established standard were reextracted, and those that still had an inadequate profile were discarded from subsequent analyses.

In this sense, of the 270 participants obtained, we were not successful in genotyping some samples, of which only one sample from the responder group was not genotyped for locus A. For locus B, 29 samples from the responder group and 31 samples from the nonresponder group were not genotyped. For the DRB1 locus, only three samples were not genotyped from the nonresponder group.

Loci A, B and DRB1 of HLA classes 1 and 2 were genotyped *via* PCR-SSO (polymerase chain reaction-sequence specific oligonucleotide) methodology using Luminex technology (Luminex Corporation, Austin, TX, USA) and a LABType^®^ kit (One Lambda Inc., Canoga Park, CA, USA).

In the amplification step, the target DNA was amplified by conventional PCR in a reaction mixture containing specific primers for each locus provided by the kit, recombinant Taq (Invitrogen, USA) and D-mix solution provided in the kit. Amplification occurred in a Mastercycler^®^ Thermal Cycler, following the amplification protocol described in the kit. To confirm the amplification, 2.5% agarose gel electrophoresis was performed.

In the hybridization stage, the amplified DNA was denatured and hybridized with a set of specific oligonucleotide probes, which were immobilized in fluorescently labelled polystyrene microspheres. After the hybridization step, the microspheres were washed and subsequently incubated with a streptavidin-phycoerythrin conjugate (SAPE), which binds to biotinylated amplified DNA.

In the last phase, a Luminex analyzer was used to measure the intensity of phycoerythrin fluorescence in each microsphere, and the data generated were stored to specific extensions. From those data, HLA Fusion software was used to determine the alleles of the HLA genes. The pattern of reactivity of each DNA sample relative to the set of probes conjugated to the microspheres allowed the establishment of the HLA genotype at medium resolution.

### Statistical Analysis

To corroborate the proposed groupings, we applied discriminant multivariate analysis that adopted the same definition criteria for the groups of nonresponders and responders; a scatter plot was inferred for group identification.

We applied Cochran’s Q test in the paired comparisons of lymphocyte quantification and viral load data obtained during the study; for intergroup analyses, we applied the Kruskal–Wallis test. We chose nonparametric tests due to the degree of normality of the variables studied, as estimated by the Lilliefors test.

For each HLA locus, Hardy-Weinberg equilibrium was measured, selecting as an alternative hypothesis of interest the excess heterozygotes in the groups; the standard parameters of the Markov chain were adopted. The genotypic linkage disequilibrium between the loci was determined by the log probability ratio, also adopting the Markov standard parameters. Allele clusters Bw4 and Bw6 were identified based on the HLA nomenclature database (34).

The parametric proportion (π) of the most frequent allele of each locus was calculated to estimate the confidence interval of the expected global frequency. We applied multivariate cluster analysis to cluster the populations of different ethnic profiles based on allele frequency data available in the “The Allele Frequency Net Database” (35). The allele frequencies of the Caucasoid, Negroid, Asian, Amerindian and heterogeneous populations of different regions of Brazil were collected; the clusters were inferred using the Ward minimum variance clustering method, with Euclidean distance and standardization of the variables.

The expected heterozygosity (*He*) for each HLA locus in the studied population was calculated based on the formula adapted from Nei et al. (36).

The frequency of the HLA alleles was calculated by direct counting and compared between the groups by the G test. For groups with significantly different allelic profiles, the chi-square residual test was applied to determine the probabilistic importance of each of the alleles for each group, followed by the calculation of the odds ratio to measure the advantage or disadvantage of the significant alleles. The comparison of the viral load and lymphocyte count data between the alleles was also performed using Cochran’s Q test for paired analysis and the Kruskal–Wallis test for interallelic analyses. Regarding ART, the rates of maintenance of treatment gains between alleles were compared using the G test.

We estimated survival curves using the Kaplan–Meier product-limit method, followed by the log-rank method (Mantel-Cox) to compare the proposed curves. We adopted the proportion of responders to ART as an event of interest in the analysis the 24-month study period as the time variant. The estimates of response to therapy were evaluated among the alleles associated with the response progression profile.

We calculated the Spearman coefficient using a general correlation matrix for all the genetic, immunological, virological and therapeutic factors evaluated. The correlations were categorized as follows: negative and strong when the Spearman coefficient was between -1.0 and -0.5; negative and weak when the Spearman coefficient was between -0.5 and 0; positive and weak when the Spearman coefficient was between 0 and 0.5; and positive and strong when the Spearman coefficient was between 0.5 and 1.0.

For the statistical analyses, we adopted a significance level (α) of 95%, considering a probability of significance (p) less than or equal to 0.05 as a criterion for rejecting the null hypotheses. The estimation of Hardy-Weinberg equilibrium and linkage disequilibrium were calculated in GENEPOP software (37). The other analyses were performed using GraphPad Prism 8.4.3 (San Diego, CA, USA) and BioEstat 5.3 (38).

## Results

### Sample Characterization

**Figure 1:**
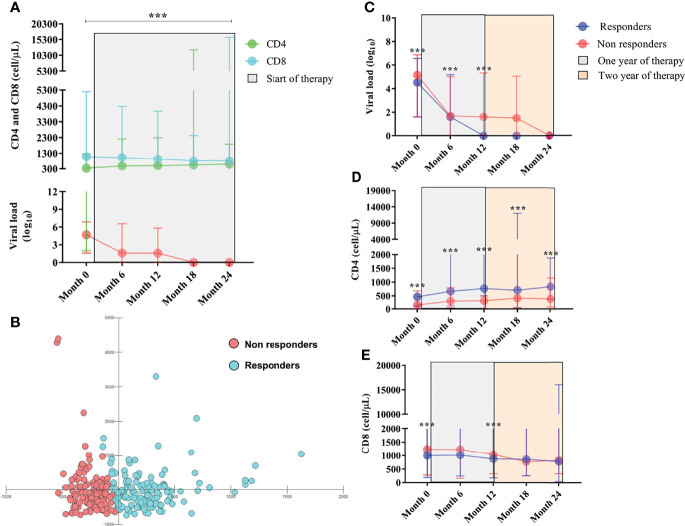
**(A)** Longitudinal immunovirological evaluation of PLHIVs before and throughout ART during the two-year treatment period. Viral load is in red. **(B)** Scatter plot of multivariate discriminant analysis of viral load and CD4+ T and CD8+ T lymphocyte counts showing the groups of therapeutic responders and nonresponders categorized based on CD4+ T lymphocyte count ≥ or < 500 cells/µL. **(C)** Longitudinal evaluation of viral load. **(D)** CD4+ T lymphocyte count and **(E)** CD8+ T lymphocyte count between responders and nonresponders before and after the start of therapy. ***: p < 0.0001.

Examining the overall infection scenario, we observed a significant reduction in HIV viral load log_10_ (*p*< 0.0001), a significant reduction in CD8^+^ T lymphocyte count (*p*< 0.0001), and an absolute increase in CD4^+^ T lymphocytes (*p*< 0.0001) after starting ART, over 24 months of observations (**Table 1**; **Figure 1A**).

**Table 1:** Analysis of HIV viral load (log10), CD4+ T lymphocyte count and CD8+ T lymphocyte count over the study period.

TIME	month 0	month 6	month 12	month 18	month 24
BIOMARKERS	Median (IQR (25%-75%)	Median (IQR (25%-75%)	Median (IQR (25%-75%)	Median (IQR (25%-75%)	Median (IQR (25%-75%)
**Overall infection scenario**					
HIV viral load (log_10_)	4.7 (4.04-5.26)	1.6 (0-1.88)	1.6 (0-1.6)	0 (0-1.6)	0 (0-1.60)
CD8^+^ T lymphocyte	1077 (758-1464)	1029 (747-1410)	926 (656-1273)	813 (609-1168)	819 (628-999)
CD4^+^ T lymphocyte	334 (169-506)	477 (306-693)	498 (322-762)	546 (373-767)	591 (384-860)
**Responders *vs* Nonresponders**					
(res.) HIV viral load (log_10_)	4.52 (3.83-4.97)	1.6 (0-1.6)	1.6 (0-1.7)	0 (0-1.6)	0 (0-1.6)
(nonres.) HIV viral load (log_10_)	4.97 (4.47-5.54)	1.6 (0-2.14)	0 (0-1.6)	0 (0-1.6)	0 (0-1.6)
(res.) CD8^+^ T lymphocyte	1232 (871-1638)	1029 (758-1452)	1037 (736-1357)	780 (583-1033)	828 (650-1028)
(nonres.) CD8^+^ T lymphocyte	1004 (715-1310)	1016 (769-1322)	878 (599-1156)	858 (640-1228)	781 (590-962)
(res.) CD4^+^ T lymphocyte	462 (350-629)	666 (540-859)	764 (612-917)	704 (571-880)	834 (620-1075)
(nonres.) CD4^+^ T lymphocyte	169 (80-280)	313 (233-400)	323 (232-400)	415 (286-528)	392 (317-580)
**Associated alleles**					
HIV viral load (log_10_)					
B*13	4.97 (4.77-5.71)	1.76 (1.2-2.43)	1.6 (1.6-2.48)	1.6 (1.2-2.47)	1.6 (1-3.4)
B*35	4.8 (4.15-5.28)	1.6 (0-1.65)	1.6 (0-1.6)	0 (0-1.6)	0 (0-1.6)
B*39	5.1 (4.48-5.55)	1.65 (1.6-2.16	1.6 (0-1.74)	0 (0-1.6)	0 (0-1.6)
Others	4.69 (4.03-5.35)	1.6 (0-1.84)	0 (0-1.6)	0 (0-1.6)	0 (0-1.6)
CD8^+^ T lymphocyte					
B*13	972 (262-985)	932 (646-1225)	745 (541-1214)	878 (521-1383)	788 (714-1304)
B*35	1110 (736-1593)	1042 (780-1459)	901 (662-1232)	787 (637-1196)	749 (596-896)
B*39	785 (559-1183)	786 (611-1400)	888 (664-1406)	902 (662-1130)	926 (538-1206)
Others	1076 (773-1520)	1029 (755-1520)	984 (672-1277)	832 (640-962)	767 (592-1024)
CD4^+^ T lymphocyte					
B*13	77 (51-314)	246 (137-340)	272 (134-407)	337 (145-562)	307 (192-457)
B*35	346 (139-512)	473 (323-789)	390 (328-697)	533 (349-709)	679 (383-928)
B*39	248 (103-446)	268 (176-400)	363 (282-599)	391 (292-664)	560 (384-614)
Others	326 (161-503)	521 (292-675)	556 (352-827)	548 (397-753)	581 (399-843)

res., responders; nonres., nonresponders; IQR, Interquartile range.

After 12 months of ART, 134 PLHIVs had a satisfactory immune response (responders), and 136 manifested failures in the restoration of CD4^+^ T lymphocyte counts (nonresponders). The multivariate analysis clearly showed that responders and nonresponders formed two distinct groups with unique immunological characteristics that did not overlap (**Figure 1B**).

In this context, the viral load log_10_ remained significantly detectable in nonresponders up to 12 months after initiating ART [(month 0 = *p*< 0.0001), (month 6 = *p*: 0.0008), (month 12 = *p*: 0.0016)] (**Table 1**; **Figure 1C**); the median CD4^+^ T lymphocyte count in nonresponders remained less than 500 cells/µL regardless of their time receiving ART [(month 0 = *p*< 0.0001), (month 6 = *p*< 0.0001), (month 12 = *p*< 0.0001), (month 18 = *p*< 0.0001), (month 24 = *p*< 0.0001)] (**Table 1**; **Figure 1D**); and we observed a tendency for CD8^+^ T lymphocyte count to be higher in nonresponders up to the first 12 months after initiating ART [(month 0 = *p*: 0.007), (month 12 = *p*: 0.0008)] (**Table 1**; **Figure 1E**).

Regarding the epidemiological, sociodemographic and therapeutic factors associated with the therapeutic response, we found that females [p = 0.037; OR (95% CI): 1.94 (1.06-3.54)], those with low education levels [p = 0.042; OR (95% CI): 1.71 (0.96-3.07)] and heterosexual individuals [p = 0.005; OR (95% CI): 2.36 (1.39-4.02)] had an approximately two times higher risk of nonresponse to ART and that an active sex life was a more frequent factor in responders [p = 0.017; OR (95% CI): 0.54 (0.33-0.89)]. A switch in treatment was the most prominent factor in the first analysis, being approximately 10 times more likely to occur in patients with immune failure after ART [p < 0.0001; OR (95% CI): 9.64 (3.66-25.43)] (**Table 2**).

**Table 2:** Epidemiological, sociodemographic and therapeutic factors associated with nonresponse to ART in PLHIVs.

	Responders n: 134	Nonresponders	*p*	OR^&^	OR^$^
FACTORS		n: 136		CI (95%)	CI (95%)
**Sex**					
Female	21 (15.67)	36 (26.47)	0.037*	1.94	
Male	113 (84.33)	100 (73.53)		(1.06-3.54)	
**Age**					
18-30	85 (63.43)	73 (53.68)	0.110*		
31-72	49 (36.57)	63 (46.32)			
**Education**					
Not literate to basic education	98 (73.13)	112 (82.35)	0.042*	1.71	
High school to higher education	36 (26.87)	24 (17.65)		(0.96-3.07)	
**Family income**					
No income to 3 minimum monthly wages	122 (91.04)	121 (88.97)	0.686*		
4 to > 10 minimum monthly wages	12 (08.96)	15 (11.03)			
**Alcohol use**					
No	12 (08.96)	17 (12.50)	0.433*		
Yes	122 (91.04)	119 (87.50)			
**Illicit drugs**					
No	94 (70.15)	100 (73.53)	0.589*		
Yes	40 (29.85)	36 (26.47)			
**Time of use**					
0 to 6 years	23 (57.50)	15 (41.67)	0.258*		
7 to more years	17 (42.50)	21 (58.33)			
**Sexual orientation**					
Heterosexual	42 (31.34)	69 (50.74)	0.005^#^	2.36	1.70
Homosexual	69 (51.49)	48 (35.29)		(1.39-4.02)	(1.00-2.89)
Bisexual	23 (17.16)	19 (13.97)			
**Active sex life**					
Yes	94 (70.15)	76 (55.88)	0.017*	0.54	
No	40 (29.85)	60 (44.12)		(0.33-0.89)	
**Permanent partner**					
No	65 (48.51)	64 (47.06)	0.903*		
Yes	69 (51.49)	72 (52.94)			
**Sexual relations with sex workers**					
No	104 (77.61)	103 (75.74)	0.774*		
Yes	30 (22.39)	33 (24.26)			
**Use of condoms**					
Occasionally	76 (56.72)	75 (55.15)	0.686^#^		
Never	15 (11.19)	20 (14.71)			
Always	43 (32.09)	41 (30.15)			
**Treatment abandonment**					
No	0	2 (01.00)	0.498*		
Yes	134 (100.0)	134 (99.00)			
**Treatment switch**					
No	129 (96.27)	99 (72.79)	<0.0001*	9.64	6.42
Yes	5 (03.73)	37 (27.21)		(3.66-25.43)	(2.12-19.39)

*Fisher’s exact test; ^#^Chi-square test; ^&^Simple logistic regression; ^$^Multiple logistic regression.

Based on the multiple logistic regression results, sexual preference [OR (95% CI): 1.70 (1.00-2.89)] and switch in therapy [OR (95% CI): 6.42 (2.12-19.39)] were the factors that remained associated with response failure (**Table 2**).

### Allelic Frequency of Loci A, B and DRB1 in the Studied Population

**Figure 2:**
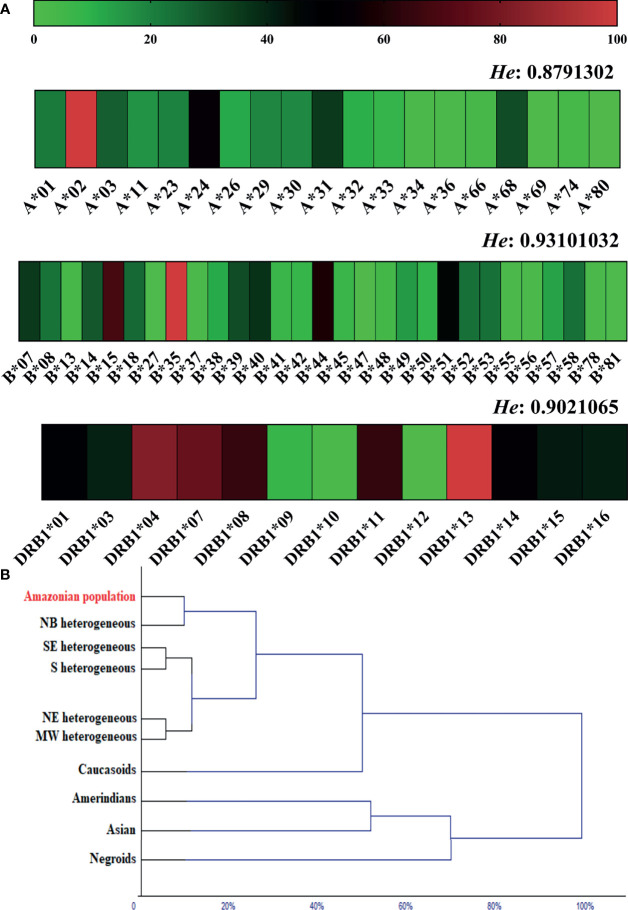
**(A)** Heatmap of the allelic frequency and expected heterozygosity (*He*) of loci A, B and DRB1 in the general population. **(B)** Dendrogram of the multivariate cluster analysis showing proximity due to allele frequency similarity of the studied population (Amazonian population) with other ethnic groups. NB, Northern Brazil; NE, Northeast Brazil; MW, Brazilian Midwest; SE, Southeast Brazil; S, Southern Brazil.

For locus A, the A*02 allele was the most common in the studied population (25.9%; 95% CI: 23.3% < π < 28.5%); for locus B, the B*35 allele was the most common (15.2%; 95% CI: 12.7% < π < 17.8%); and for the DRB1 locus, the DRB1*13 allele was the most common (14.8%; 95% CI: 12.7% < π < 16.9%). The expected heterozygosity was highest for locus B (*He*: 0.93) (**Figure 2A**). All loci were in Hardy-Weinberg equilibrium (locus A, p = 0.777; locus B, p = 0.896; and locus DRB1, p = 0.974). All loci were in linkage equilibrium; therefore, it was assumed that the segregation of the allele pairs was random (locus AB, p = 1.000; locus A-DRB1, p = 0.220; and locus B-DRB1, p = 0.300).

In the cluster analysis, the allelic frequencies of loci A, B and DRB1 in the studied population (Amazon population) were similar to those observed in heterogeneous ethnic populations from different regions of Brazil, more specifically from northern Brazil. The allele frequency for Caucasoids was directly related to the heterogeneous clade; in another segment, we observed a closer relationship between Amerindians and Asians; and Negroes were related to this clade at the second level (**Figure 2B**).

**Figure 3:**
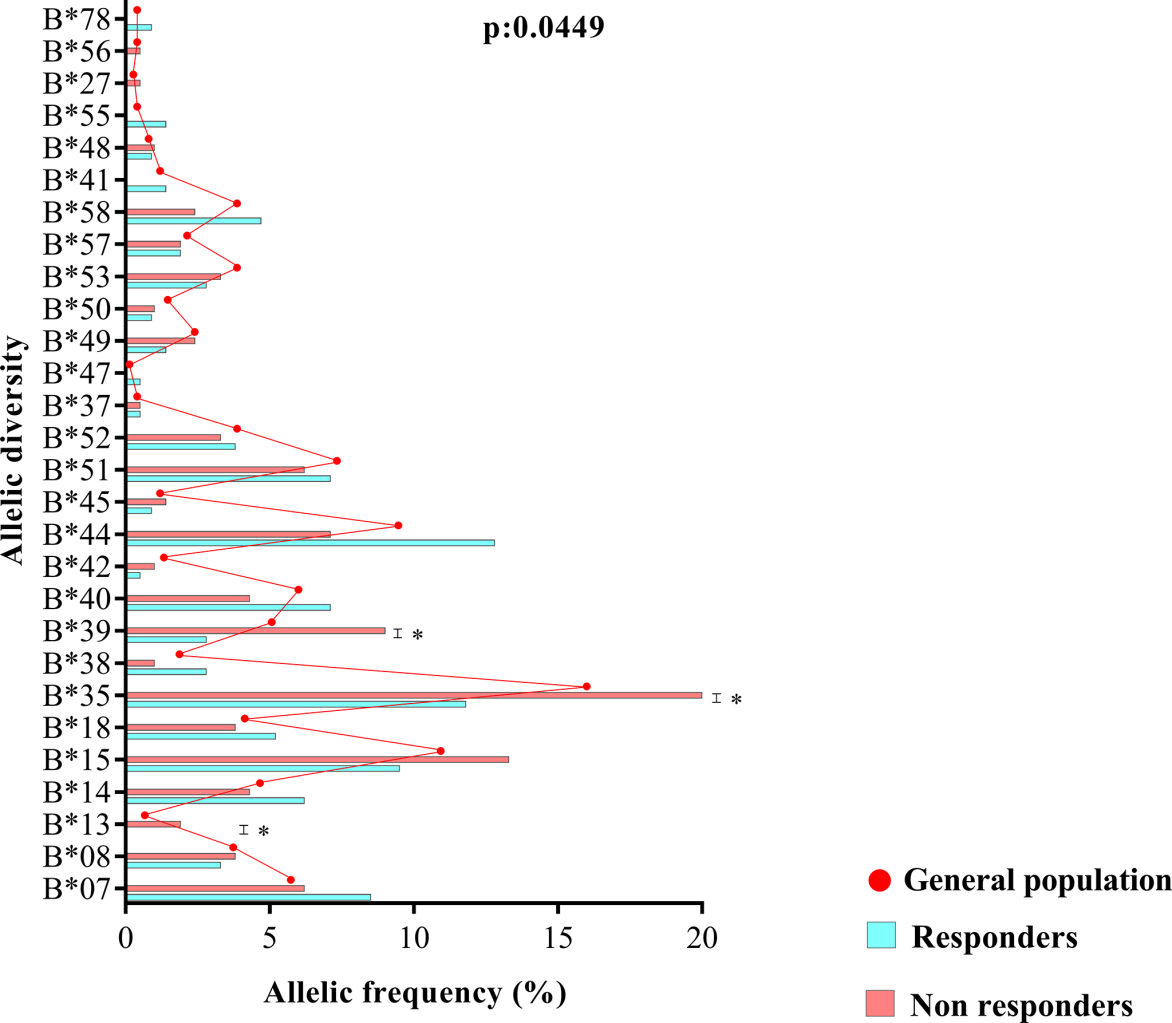
Allele diversity of the HLA-B locus between treatment responders, treatment nonresponders and the entire study population (general population). The B*13, B*35 and B*39 alleles were more common in nonresponders (p = 0.0449).

The allelic frequencies of loci A and DRB1, as well as their zygosity, were similar between ART responders and nonresponders (locus A, p = 0.9319; zygosity, p = 0.3561); (locus DRB1, p = 0.1516; zygosity, p = 0.9817) (**Supplementary Table 1**). However, the allelic profile of locus B was statistically associated with therapeutic nonresponse (p = 0.0449) (**Figure 3**). Based on the chi-square residual, alleles B*13, B*35 and B*39 had the greatest probabilistic influence in the analysis; both zygosity (p: 0.6760) and the frequency of clusters Bw4 and Bw6 (p: 0.9386) were not associated with the profile (**Supplementary Table 1**).

### The B*13, B*35 and B*39 Alleles Were Associated With Immunological Aspects of the Therapeutic Response

**Figure 4:**
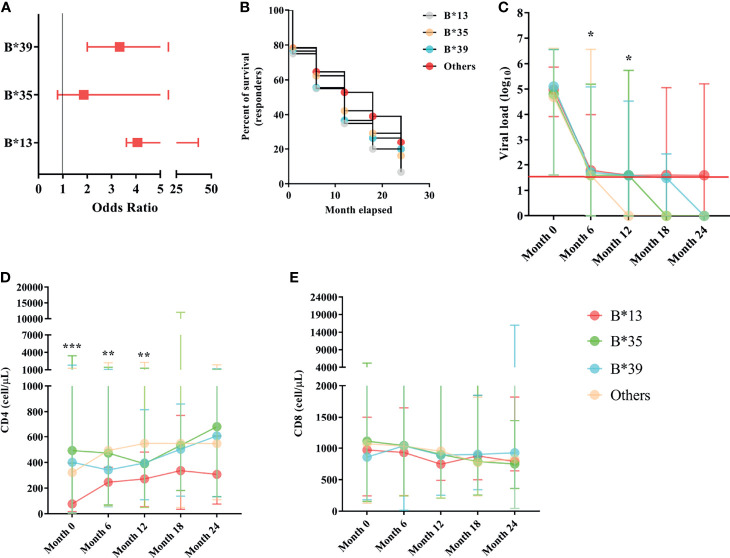
**(A)** Box plot graph showing the odds ratio values for the risk of therapeutic nonresponse associated with the B*13, B*35 and B*39 alleles. The horizontal bars are the statistical confidence interval for each odds ratio. **(B)** Kaplan–Meier and Mantel-Cox survival curve models for alleles B*13, B*35, B*39 and other alleles related to the proportion of ART responders across 24 months of observation; the proposed curves were significant (x^2^ = 9.923; p = 0.0192); the B*13 allele occurred in the lowest proportion of responders over the study period (6.67%). **(C)** Longitudinal evaluation of viral load. The red line represents the detection limit of the kit used. **(D)** CD4+ T lymphocyte count and **(E)** CD8+ T lymphocyte count among carriers of alleles B*13, B*35, B*39 and other alleles in the 24-month period; ***: p < 0.0001; **: 0.005 ≥ p < 0.0001; *: 0.05 ≤ p > 0.0001.

In the odds ratio calculation, the B*13, B*35 and B*39 alleles were associated with the risk of therapeutic nonresponse in PLHIVs, and the odds ratio for nonresponse was approximately twofold for the B*35 allele [OR (95% CI): 1.86 (1.08-3.18)] and threefold for the B*39 allele [OR (95% CI): 3.33 (1.33-8.69)]; the B*13 allele, with the highest ratio, had an odds ratio for nonresponse approximately fourfold higher [OR (95% CI): 4.06 (0.45-36.62)]. We suggest that the extensive confidence interval is a reflection of the low allele frequency in the groups (**Figure 4A**). The estimated survival curves were significant for the comparison methods used (x^2^ = 9.923; p = 0.0192); the B*13 allele had the lowest proportion of ART responders across the study period, with approximately 6.67% of responders at the end of 24 months of analysis, followed by the B*35 allele (16.15%), B* 39 (19.93%) and other alleles (24.05%) (**Figure 4B**).

The viral load was similar among the B locus alleles in the pretreatment collection; however, a threshold of statistical significance was observed at six months of therapy, with carriers of the B*13 allele having the highest viral load (*p*: 0.0526; **Table 1**). A statistically significant association was observed after 12 months of therapy, during which only carriers of alleles without risk of therapeutic response (other alleles) maintained viral loads below the limit of detection (p = 0.0237; **Table 1**). After 12 months of therapy, the viral load for all alleles was statistically similar and close to the limit of detection; carriers of the B*35 allele had very low viral loads at 18 months of therapy, and carriers of the B*39 allele had very low viral loads at 24 months; however, carriers of the B*13 allele still had detectable viral loads even with a long period of treatment (**Table 1**; **Figure 4C**).

The CD4^+^ T lymphocyte count remained statistically low in the B*13 allele carriers through the first 12 months of observation [(month 0 = *p*< 0.0001), (month 6 = *p*: 0.017), (month 12 = *p*: 0.0032)]. After 12 months of ART, the lymphocyte count was standardized among the alleles; however, the carriers of the B*13 allele had low counts relative to the others (**Table 1**; **Figure 4D**).

The number of CD8^+^ T lymphocytes was similar among the alleles, regardless of the duration of ART (**Figure 4E**). In a longitudinal analysis, there was a reduction in cellularity in carriers of the B*35 allele (*p*: 0.005) and carriers of alleles without a response risk (*p*< 0.0001) over the 24 months of therapy (**Table 1**).

### The B*13 Allele May be Associated With Therapy Switching

**Figure 5:**
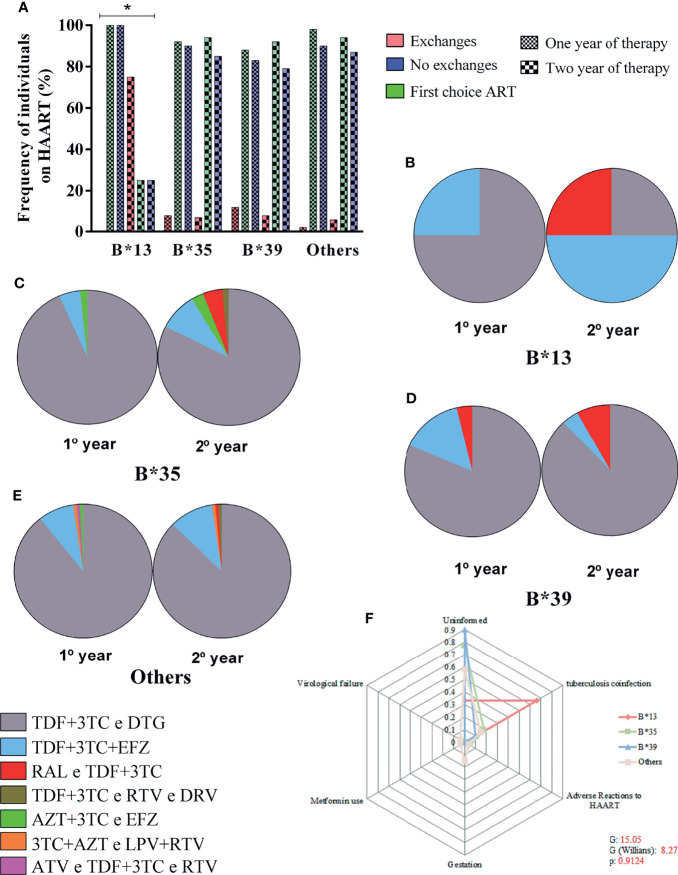
**(A)** Frequency plot of the rate of maintenance of therapeutic regimens among carriers of alleles B*13, B*35, B*39 and other alleles in the 24-month period; the treatment switching rate was associated with allele B*13; *: 0.05 ≤ p > 0.0001. **(B–E)** Diversity of therapeutic regimens administered in 1- and 2-year periods in carriers of alleles B*13 **(B)**, B*35 **(C)**, B*39 **(D)** and other alleles **(E)**. **(F)** Radar chart showing the reasons for switching therapy between carriers of alleles B*13, B*35, B*39 and other alleles; each equiangular spoke represents a reason for switching therapy; approximately 66% of B*13 allele carriers switched treatments due to coinfection with tuberculosis. TDF, tenofovir; 3TC, lamivudine; DTG, dolutegravir; EFZ, efavirenz; RAL, raltegravir; RTV, ritonavir; DRV, darunavir; AZT, zidovudine; LPV, lopinavir.

We evaluated the frequency of ART replacement in the intervals of 0-12 months and 12-24 months of treatment, for which we calculated the rate of regimen switching, the rate of maintaining the previous regimen and the rate of continuing the first therapeutic regimen since the beginning of monitoring. After 24 months of therapy, there was a significant 75% (3 to 4) increase in the rate of regimen switching among carriers of the B*13 allele and a 25% decrease (1 to 4) in the rates of maintaining and continuing previous regimens (*p*: 0.0344; **Figure 5A**). However, we detected only four carriers of the allele in the present study, which limits more robust conclusions about the relationship between genotype and maintenance of therapy. We describe here only one observed trend.

In the first 12 months of therapy, 75% of carriers of the B*13 allele were treated with the tenofovir + lamivudine and dolutegravir regimen (TDF+3TC and DTG), and only 25% started treatment with the second most common regimen: tenofovir + lamivudine + efavirenz (TDF+3TC+EFZ). However, after 24 months of therapy, we observed an inversion of prevalence, with the TDF+3TC+EFZ regimen becoming the most common (50%); the TDF+3TC and DTG regimen was maintained for 25% of patients, and a new regimen, raltegravir and tenofovir + lamivudine (RAL and TDF+3TC), was administered to 25% of patients (**Figure 5B**). For the other locus B alleles, the TDF+3TC and DTG regimen prevailed regardless of the duration of therapy, with a frequency ranging from 81% to 93% (**Figures 5C–E**).

The reasons for switching therapy were not reported for most patients (50-90%); approximately 66% of carriers of the B*13 allele switched due to coinfection with tuberculosis, and approximately 5% of carriers of the B*35 allele switched therapy due to adverse reactions to the previous regimen. Pregnancy (15%), use of metformin as an antidiabetic agent (4%) and virological failure (4%) were reported only for carriers of other alleles. The frequency of the reasons for switching was not significant among the alleles (G: 15.05; G (Williams): 8.27; *p*: 0.9124) (**Figure 5F**).

### General Correlation Matrix

**Figure 6:**
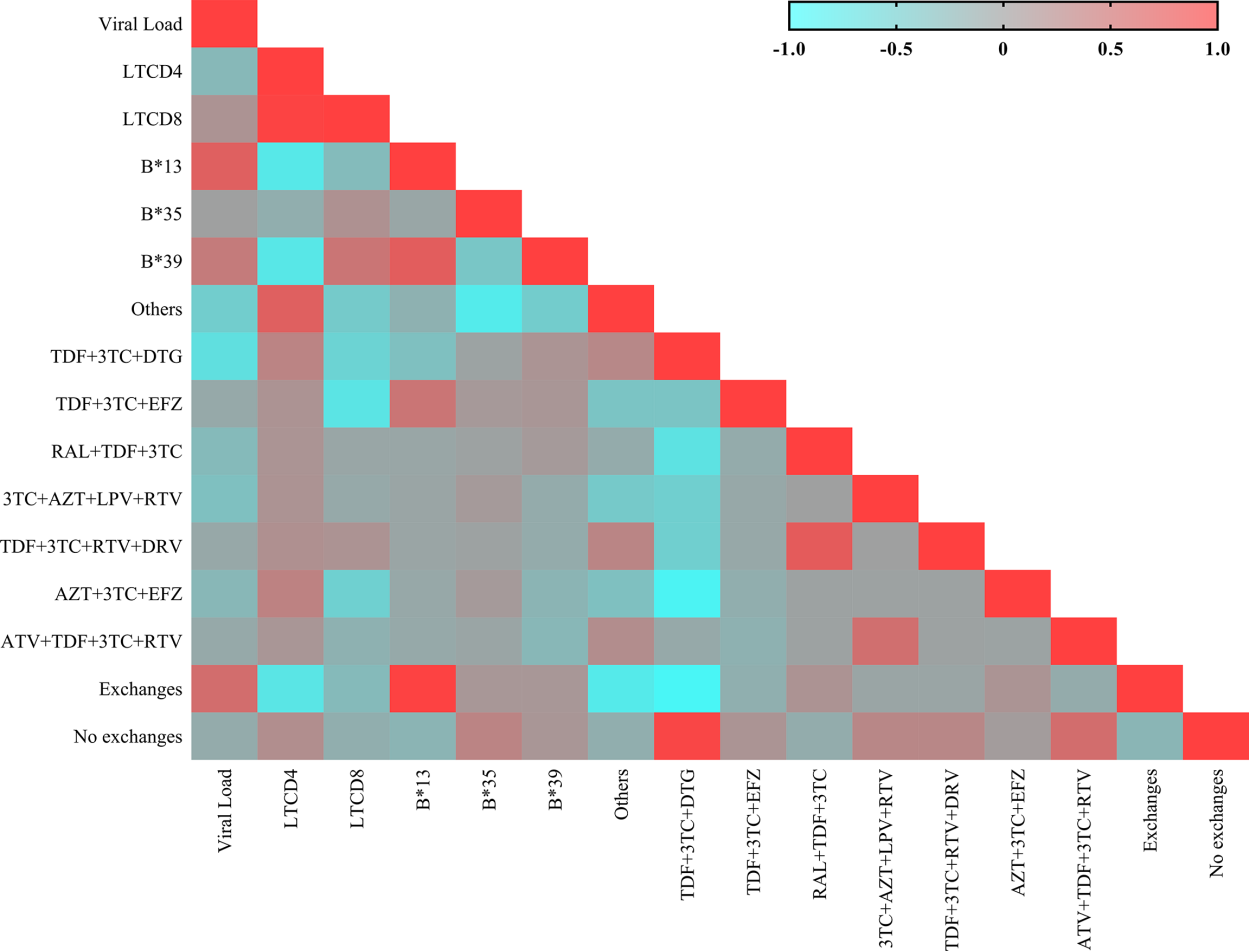
Heatmap showing the matrix of correlations between the immunovirological and genetic variables. Each square represents the Spearman coefficient of the correlation between two variables. Negative and strong correlations have a coefficient between -1.0 and -0.5; negative and weak correlations have a coefficient between -0.5 and 0; positive and weak correlations have a coefficient between 0 and 0.5; and positive and strong correlations have a coefficient between 0.5 and 1.0.

We generated a correlation matrix for the genetic, immunological and therapeutic factors evaluated in the present study (**Figure 6**).

The B*13 allele was significantly correlated with switching therapy (r: 0.975; *p*: 0.0210), the TDF+3TC+EFZ regimen (r: 0.451; *p*: 0.0394), a low CD4^+^ T lymphocyte count (r: -0.765; *p*: 0.0265) and increased plasma viral load (r: 0.662; *p*: 0.0546). The B*35 allele was not correlated with the factors evaluated. The B*39 allele was correlated with an increased viral load (r: 0.440; *p*: 0.0379), a low CD4^+^ T lymphocyte count (r: -0.747; *p*: 0.0304) and a high CD8^+^ T lymphocyte count (r: 0.440; *p*: 0.0452).

The therapeutic regimens significantly correlated with reductions in viral load were TDF+3TC and DTG (r: -0.667; *p*< 0.0001) and TDF+3TC+EFZ (r: -0.109; *p*: 0.0016). The maintenance of the CD4^+^ T lymphocyte count was correlated only with the TDF+3TC and DTG regimen (r: 0.191; *p*< 0.0001). A reduction in CD8^+^ T lymphocyte count was correlated with the TDF+3TC+EFZ regimen (r: -0.071; *p*: 0.0385). Most patients administered the RAL+TDF+3TC (r: 0.135; *p*: 0.0001) and AZT+ 3TC+EFZ (r: 0.107; *p*: 0.0020) regimens switched therapy during the two years of treatment. The TDF+3TC and DTG (r: 0.923; *p*: 0.0004) and TDF+3TC+EFZ (r: -0.119; *p*: 0.0006) regimens were correlated with maintaining the same regimen throughout the two years of treatment.

## Discussion

In the present study, 50.37% of the participants were treatment nonresponders, a value within the expected range based on the heterogeneity of studies on the evaluation of suboptimal immune recovery (39).

The duration of treatment required to categorize therapeutic response is also a contrasting factor (40). We suggest that with two years of therapy, the classification of groups was feasible due to the noticeable delineation of the response status. Reports indicate that between one and three years of continuous therapy, the evaluation of biomarkers associated with therapy is functionally able to stratify groups regarding therapeutic response (2, 41, 42).

Among the set of associated factors, the genetic profile of key elements in the immune response may also influence recovery failure (5). Interestingly, our results showed that even before the onset of ART, some markers of infection progression were already different among patients who would be classified based on therapeutic response. This fact supports the hypothesis that hereditary factors play a role in possible susceptibility to failure of immune reconstitution. We validated this hypothesis by demonstrating that the HLA class 1 B*13, B*35 and B*39 alleles were associated with nonresponders.

Herein, we demonstrated that the B*35 allele was the most prevalent allele in the general population and was associated with the lowest risk of therapeutic response failure; however, the proportion of responders after two years of therapy was approximately 16%. Carriers of the allele maintained a detectable viral load up to 12 months after the initiation of therapy, with a decrease in the CD4^+^ T lymphocyte count during the same period. The B*35 allele is in fact consistently associated with the progression of HIV infection because CD8^+^ T lymphocytes associated with this allele exhibit a dysfunctional phenotype that cannot elicit an effective response to control viral replication (43); essentially, we found that the concentration of CD8^+^ T lymphocytes decreased in B*35 allele carriers in the two years of observation. The immune response by B*35+ cells may be robust due to avidity in antigen presentation; however, this may be influenced by epitope type or the dominant viral clade (44, 45).

Subvariants of the B*35 allele that affect antigen presentation are associated with different outcomes regarding the risk of HIV progression (46, 47). In our study, the genotyping resolution allowed us to identify only the allele group of each locus and was not specific enough to distinguish other levels of classification. Therefore, the grouping of functionally distinct variations may have influenced the nonpresentation of more categorical associations of the risk allele with the pathological markers.

Carriers of the B*39 allele were approximately three times more likely to present therapeutic response failure. The viral load remained detectable after 18 months of continuous therapy; however, there was an increase in the CD4^+^ T lymphocyte count after 12 months of ART and a positive correlation with CD8^+^ T lymphocytes. This attempt at an immune rebound is consistent with reports that show an association of B*39 with slower disease progression due to epitopes with fitness costs to the virus (48, 49). A possible justification is that the cellular response restricted to the allele tends to be more robust and substantial, resulting in improved effector activity with impacts on clinical aspects (50, 51).

The B*13 allele was more closely associated with the risk of response failure than were the other alleles, with approximately 7% of responders at the end of two years of therapy and a detectable viral load and CD4^+^ T lymphocyte count below 400 cells/µL over the same period. These findings contrast with most of the reports that show an association of the B*13 allele with a robust immune response, especially against epitopes originating from the Gag and Nef regions, given that the restricted mutations incur a significant viral fitness cost (52–54). However, the claimed strong effect on disease control is more noticeable in the long term (55) and is probably masked by the noncontinuous administration of antiretrovirals, even for brief periods (56). In contrast, the association of the B*13 allele with mutational resistance variants that hinder immunological recognition and favor therapeutic switches in the short and medium terms has been discussed (57, 58).

In this regard, the treatment switch rate was higher in carriers of the B*13 allele in the second year of treatment, and the allele was correlated with the maintenance of the TDF+3TC+EFZ regimen. According to Brazilian guidelines, for patients beginning treatment, the preferred regimen is a combination of nucleoside reverse transcriptase inhibitors (NRTI), 3TC+TDF, and the integrase inhibitor (INI) DTG (59). Users of 3TC+TDF have a favorable profile regarding virological suppression and CD4^+^ T lymphocyte response (60); it is an interchangeable regimen with low rates of relative risk and few resistant strains (61) that can also be administered in cases of hepatitis B coinfection (62). The addition of DTG provides a high genetic barrier with few adverse events, especially for treatment-free PLHIVs (63).

However, the exception to this preferred regimen is coinfection with tuberculosis, for which the administration of a nonnucleoside reverse transcriptase inhibitor (NNRTI), EFZ, is indicated as a substitute for DTG (59) because the treatment of tuberculosis can alter the effective concentration of DTG in the body (64). The pharmacological interaction of EFZ with anti-tuberculosis agents is associated with successful virological results (65). This is consistent with our results, i.e., the regimen is correlated with the control of viral load and maintenance of the CD8^+^ T lymphocyte count.

Studies have discussed the association of the B*13 allele with the risk of developing thoracic tuberculosis and multisystemic sarcoidosis (66, 67). Interestingly, in the present study, we also showed that 50% of B*13 allele carriers who switched treatment regimens in the second year of treatment were coinfected with tuberculosis.

Thus, our dataset suggests that at the beginning of HIV infection, the B*13 allele may be favored after an attempted antiviral response, even in patients undergoing intensive therapy and showing a sustained virological response, which predispose these patients to coinfections that require changes to specific therapeutic regimens. This could be one of the causes of the high incidence of tuberculosis in nonresponders and the direct relationship with CD4^+^ T count, regardless of virological status (68, 69). In addition, HLA can mediate the loss of CD4^+^ T lymphocytes in nonresponders due to the unregulated immune activation in HIV infection, even though ART is being administered (70).

Our findings on the association of the B*13 allele with therapeutic nonresponse, although intriguing, require further investigation, as our sample size may not be satisfactory for accurate conclusions.

This is the first study to associate HLA allele variants with the therapeutic response status in PLHIVs in the Brazilian Amazon region. Our findings indicate that the allele frequencies of HLA-I and HLA-II were similar to those for heterogeneous populations, especially in the northern region of the country, a finding that was expected given the diversified ethnic influence on regional population structure (71). In addition, we noted a similarity in the allele frequencies of Caucasoids with the population studied, a finding also observed in other studies of Amazon ancestry profiles (72). The analyses herein contribute to studies of HLA associations on intra- and interethnic scales and validate our proposal and those of others (73) specifically for HIV infection, i.e., pathological diversification is a continuous process that is influenced by the genetic profile of the ethnic composition of an infected population (74).

Illiterate and heterosexual women were more common in the group of nonresponders; however, we are unsure of the scientific implications of sociodemographic profile in the immune reconstitution of patients. Future studies on that issue may establish whether these findings may in fact be associated with an immunovirological response (75) or specific viral transmissibility routes (76) or a reflection of the social epidemiology of HIV (77).

We conclude that the allelic variants of the B locus are more associated with nonresponse to therapy in PLHIV from a heterogeneous population in the Brazilian Amazon. The sample size may have been a limiting factor in our study; however, we do believe that our findings provide a better understanding of the role of HLA in the context of the antiretroviral therapeutic response in HIV infection.

## Data Availability Statement

The original contributions presented in the study are included in the article/**Supplementary Material**. Further inquiries can be directed to the corresponding author.

## Ethics Statement

The studies involving human participants were reviewed and approved by Human Research Ethics Committee of Instituto Evandro Chagas (Protocol: 3.121.265; CAAE: 73927717.3.0000.0019). The patients/participants provided their written informed consent to participate in this study.

## Author Contributions

TAFM, OM, RCMS, FBF, IBC, and ACRV were the creators of the project. LMSP, ESF, ABCF, and FLPR were responsible for sample collection at reference centers. LMSP, ESF and IrBC developed the methodology for the project; specifically, EVOJ and PJSMM conducted the HLA loci genotyping. LMSP conducted the statistical analysis and wrote the article. ESF, FBF, IgBC, and ACRV reviewed the article. All authors contributed to the article and approved the submitted version.

## Funding

This study was funded by specific incentives from *Secretaria de Vigilância em Saúde do Ministério da Saúde* (Health Surveillance Secretariat of the Ministry of Health), *Conselho Nacional de Desenvolvimento Científico e Tecnológico* (National Council for Scientific and Technological Development; CNPQ) (no. 301869/2017-0) and Fundação Amazônia de Amparo a Estudos e Pesquisa – FAPESPA (ICAAF-60/2020). We thank *Coordenação de Aperfeiçoamento de Pessoal de Nível Superior* (Brazilian Federal Agency for the Support and Evaluation of Graduate Education; CAPES) for granting a scholarship (process number: 88882.183970/2018-01).

## Conflict of Interest

The authors declare that the research was conducted in the absence of any commercial or financial relationships that could be construed as a potential conflict of interest.

## Publisher’s Note

All claims expressed in this article are solely those of the authors and do not necessarily represent those of their affiliated organizations, or those of the publisher, the editors and the reviewers. Any product that may be evaluated in this article, or claim that may be made by its manufacturer, is not guaranteed or endorsed by the publisher.
