# Supplementary material for: HLA-B*13, B*35 and B*39 Alleles Are Closely Associated With the Lack of Response to ART in HIV Infection: A Cohort Study in a Population of Northern Brazil
Source: Front Immunol. 2022 Mar 16;13:829126. doi: 10.3389/fimmu.2022.829126 (PMC8966405; doi:10.3389/fimmu.2022.829126)
Supplement: Supplementary file 1 [file Table_1.pdf]

Supplementary Table 1- HLA class I and II allele frequencies for loci A, B and DRB1 among nonresponder and responder PLHIVs.

Alleles	Nonresponders		Responders				Nonresponders	Responders
	N	%	N	%	p	G test	x^2 Residuals (α: 0.05): 1.96	x^2 Residuals (α: 0.05): 1.96
A*01	14	5.15	14	5.26	0.9319	10.8120	-0.0606	0.0606
A*02	68	25.00	66	24.81			0.0504	-0.0504
A*03	16	5.88	14	5.26			0.3129	-0.3129
A*11	14	5.15	16	6.02			-0.4387	0.4387
A*23	16	5.88	16	6.02			-0.0651	0.0651
A*24	45	16.54	28	10.53			2.0378	-2.0378
A*26	6	2.21	9	3.38			-0.8295	0.8295
A*29	10	3.68	10	3.76			-0.0508	0.0508
A*30	14	5.15	10	3.76			0.7795	-0.7795
A*31	26	9.56	30	11.28			-0.6529	0.6529
A*32	5	1.84	8	3.01			-0.8830	0.8830
A*33	4	1.47	7	2.63			-0.9514	0.9514
A*34	1	0.37	3	1.13			-1.0262	1.0262
A*36	2	0.74	1	0.38			0.5596	-0.5596
A*66	3	1.10	2	0.75			0.4243	-0.4243
A*68	24	8.82	25	9.40			-0.2317	0.2317
A*69	0	0.00	1	0.38			-1.0122	1.0122
A*74	3	1.10	5	1.88			-0.7443	0.7443
A*80	1	0.37	1	0.38			-0.0158	0.0158
Homozygotes	14	0.10	19	0.14	0.3561	0.9983	-0.9977	0.9977
Heterozygotes	122	0.90	114	0.86			0.9977	-0.9977
B*07	13	6.19	18	8.49	0.0451	40.1008	-0.9193	0.9193
B*08	8	3.81	7	3.30			0.2723	-0.2723
B*13	4	1.90	0	0.00			2.0143	-2.0143
B*14	9	4.29	13	6.13			-0.8646	0.8646
B*15	28	13.33	20	9.43			1.2442	-1.2442

B*18	8	3.81	11	5.19			-0.6937	0.6937
B*35	42	20.00	26	12.26			2.2861	-2.2861
B*38	2	0.95	6	2.83			-1.4211	1.4211
B*39	19	9.05	6	2.83			2.6931	-2.6931
B*40	9	4.29	15	7.08			-1.2492	1.2492
B*42	2	0.95	1	0.47			0.5835	-0.5835
B*44	15	7.14	27	12.74			-1.9353	1.9353
B*45	3	1.43	2	0.94			0.4552	-0.4552
B*51	13	6.19	15	7.08			-0.3782	0.3782
B*52	7	3.33	8	3.77			-0.2536	0.2536
B*37	1	0.48	1	0.47			0.0034	-0.0034
B*47	0	0.00	1	0.47			-0.9988	0.9988
B*49	5	2.38	3	1.42			0.7207	-0.7207
B*50	2	0.95	2	0.94			0.0048	-0.0048
B*53	7	3.33	6	2.83			0.2904	-0.2904
B*57	4	1.90	4	1.89			0.0068	-0.0068
B*58	5	2.38	10	4.72			-1.3053	1.3053
B*41	0	0.00	3	1.42			-1.7341	1.7341
B*48	2	0.95	2	0.94			0.0048	-0.0048
B*55	0	0.00	3	1.42			-1.7341	1.7341
B*27	1	0.48	0	0.00			1.0036	-1.0036
B*56	1	0.48	0	0.00			1.0036	-1.0036
B*78	0	0.00	2	0.94			-1.4142	1.4142
Homozygotes	10	0.10	13	0.12	0.6760	0.4090	-0.6386	0.6386
Heterozygotes	95	0.90	93	0.88			0.6386	-0.6386
Bw4	36	0.30	33	0.30	0.9386	0.127	-0.0865	0.0865
Bw4/Bw6	57	0.47	49	0.45			0.3271	-0.3271
Bw6	28	0.23	27	0.25			-0.2894	0.2894
DRB1*01	27	10.15	24	8.96	0.1516	17.3648	0.4698	-0.4698
DRB1*03	23	8.65	15	5.60			1.3705	-1.3705

DRB1*04	38	14.29	31	11.57			0.9364	-0.9364
DRB1*07	28	10.53	36	13.43			-1.0340	1.0340
DRB1*08	28	10.53	18	6.72			1.5689	-1.5689
DRB1*09	6	2.26	5	1.87			0.3172	-0.3172
DRB1*10	5	1.88	1	0.37			1.6515	-1.6515
DRB1*11	26	9.77	30	11.19			-0.5353	0.5353
DRB1*12	5	1.88	3	1.12			0.7231	-0.7231
DRB1*13	27	10.15	44	16.42			-2.1328	2.1328
DRB1*14	26	9.77	20	7.46			0.9520	-0.9520
DRB1*15	15	5.64	23	8.58			-1.3226	1.3226
DRB1*16	12	4.51	18	6.72			-1.1065	1.1065
Homozygotes	16	0.12	17	0.13	0.9817	0.027	-0.1630	0.1630
Heterozygotes	117	0.86	117	0.87			0.1630	-0.1630
